# The Growing Evidence for the Importance of the Otoliths in Spatial Memory

**DOI:** 10.3389/fncir.2019.00066

**Published:** 2019-10-18

**Authors:** Paul F. Smith

**Affiliations:** ^1^Department of Pharmacology and Toxicology, Brain Health Research Centre, School of Biomedical Sciences, University of Otago Medical School, Dunedin, New Zealand; ^2^Brain Research New Zealand, Auckland, New Zealand; ^3^Eisdell Moore Centre for Hearing and Balance Research, University of Auckland, Auckland, New Zealand

**Keywords:** otoliths, vestibular, spatial learning and memory, hippocampus, head direction cells, place cells

## Abstract

Many studies have demonstrated that vestibular sensory input is important for spatial learning and memory. However, it has been unclear what contributions the different parts of the vestibular system – the semi-circular canals and otoliths – make to these processes. The advent of mutant otolith-deficient mice has made it possible to isolate the relative contributions of the otoliths, the utricle and saccule. A number of studies have now indicated that the loss of otolithic function impairs normal spatial memory and also impairs the normal function of head direction cells in the thalamus and place cells in the hippocampus. Epidemiological studies have also provided evidence that spatial memory impairment with aging, may be linked to saccular function. The otoliths may be important in spatial cognition because of their evolutionary age as a sensory detector of orientation and the fact that velocity storage is important to the way that the brain encodes its place in space.

## Introduction

**FIGURE 1:**
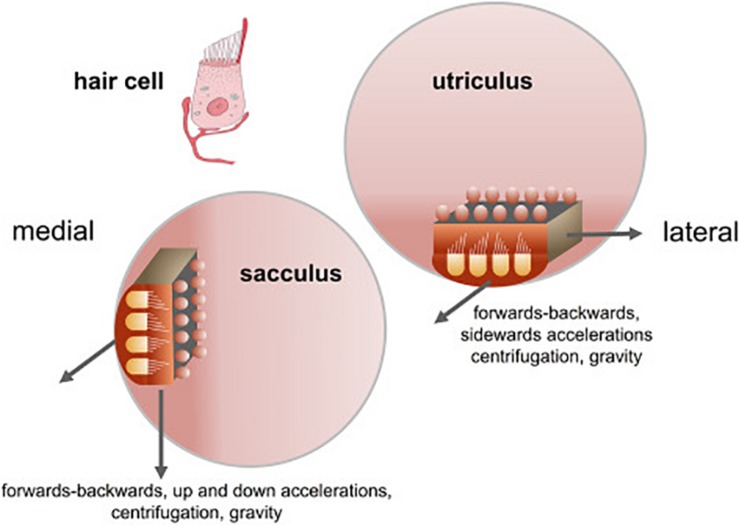
Orientation of the sacculus and utriculus in the labyrinth. From Kingma and Van De Berg (2016) with permission.

The otolith organs in the vestibular inner ear, which comprise the utricle and saccule, represent the most ancient part of the vestibular system in evolutionary terms. Estimated to have evolved more than 500 million years ago, a primitive otolithic system (“statoliths”) even exists in invertebrates such as jellyfish (see Fritzsch, 1998; Spoor et al., 2002; Jeffery and Spoor, 2004; Schulz-Mirbach et al., 2019, for reviews). In mammals, the vestibular system develops early in embryogenesis and provides the fetus with the ability to detect gravitational vertical and self-motion before birth (Baker, 1998; Ronca, 2003; Jeffery and Spoor, 2004; Ronca et al., 2008; Lim and Brichta, 2016). Unlike the visual system, the vestibular system is almost fully functional at birth (see Lim and Brichta, 2016 for a review) and provides the brain and body with an immediate sense of direction in the spatial environment (Jamon, 2014). While the three semi-circular canals detect angular acceleration of the head in different planes, the otoliths, the utricle and saccule, sense linear acceleration, including linear acceleration by gravity. The maculae of the utricle and saccule are oriented at right angles to one another, and in the usual supine position of the head for humans, the saccule responds to changes in acceleration by gravity as the head is tilted relative to gravitational vertical (see Figure 1). In both the utricle and saccule, the hair cells, which are oriented in different directions, are activated by the inertial force exerted upon them by otoconia (calcium carbonate crystals) which sit above, on an epithelial layer (see Lim and Brichta, 2016 for a review; Figure 1). The specific role of the otoliths is to provide a gravitational frame of reference with which to interpret other sensory signals, including those generated by the semi-circular canals, and to contribute to the perception of linear motion within that framework (Andreescu et al., 2005; Cullen, 2012; Jamon, 2014; Curthoys et al., 2017). This, in turn, is critical for the vestibular contribution to cognitive processes such as spatial memory (see Besnard et al., 2016; Smith, 2017 for reviews), the body representation of the self (see Mast et al., 2014; Lopez et al., 2015 for reviews), and even social cognition (see Deroualle and Lopez, 2014 for a review). Any role that the otoliths have in these processes is especially important since: aging is associated with reduced otolith function (e.g., Agrawal et al., 2012; Zu Eulenburg et al., 2017); impaired vestibular function and specifically otolithic function, has been recently associated with an increased risk of cognitive impairment and dementia (Aranda-Moreno and Jáuregui-Renaud, 2016; Harun et al., 2016; Wei et al., 2017, 2019; Xie et al., 2017; Kamil et al., 2018, in press; see Agrawal et al., 2019, in press, for a review); and otolithic lesions can occur in humans independently of lesions of the semi-circular canals (e.g., Manzari et al., 2014). In addition to other forms of dysfunction that can occur in the vestibular system (e.g., benign paroxysmal positional vertigo, vestibular migraine, Meniere’s disease, vestibular vertigo, vestibular neuritis, vestibular schwannomas, etc.), there is evidence that high intensity noise exposure can damage the otoliths as well as the cochlea (e.g., Stewart et al., 2016; Xu et al., 2016).

While evidence has continued to accumulate for a significant role of the vestibular system as a whole, in the development of spatial learning and memory, the importance of the otoliths themselves has remained relatively obscure, due to the difficulty in accessing them surgically in order to lesion or electrically stimulate them selectively and independently of the semi-circular canals, in experimental studies (e.g., Hitier et al., 2016). Hence, while there is a wealth of evidence that global lesions of the peripheral vestibular system impair spatial memory in animals and humans in a variety of tasks (e.g., Zheng et al., 2006, 2009; Baek et al., 2010; see Besnard et al., 2016; Smith, 2017, for reviews), there are relatively few data relating specifically to the otoliths. Studies in extraterrestrial microgravity and in parabolic flight are possible avenues to investigate their significance; however, in each case there are confounding factors (e.g., Zheng et al., 2017). In microgravity the otoliths are no longer stimulated by gravity, but they respond to other forms of linear acceleration (Ronca, 2003). In parabolic flight, subjects experience microgravity but they are also subjected to hypergravity, which may cancel out the effects of the microgravity (Zheng et al., 2017). One development that has provided a new path to investigate the specific contributions of the otoliths, is the production of mutant mouse strains which are devoid of otoconia and therefore, of otolithic function. The aim of this review is to summarize and critically evaluate what is currently known of the importance of the otoliths for spatial memory and its neural substrates.

## Evidence for Otolithic Involvement in Spatial Memory in Mice

There are relatively few studies to date, but one model of otolith loss is the B6Ei.GL-Nox3het/J mouse (“*tilted Het*” mouse), which presents a mutation on chromosome 17 which inhibits the expression of the NADPH oxidase 3 gene (Paffenholz, 2004). *Het–/–* mice exhibit a complete and specific loss of utricular and saccular otoconia (Bergstrom et al., 1998). Another model is the –/–; B6.Cg-*Otop1tlt/j tilted* mouse (Jackson Laboratories, Bar Harbor, ME; Jones et al., 2008; Kim et al., 2010), which exhibits a similar deletion of the otoliths (de Caprona et al., 2004; Blankenship et al., 2017; Khan et al., 2019a, b).

The importance of the otoliths for spatial memory has been demonstrated in a number of studies published between 2012 and 2019, which have tested otolith-deficient mice in behavioral tests that assess cognitive performance. Most of these have involved testing spatial memory (e.g., the Y maze test, place recognition test, radial arm maze test, Barnes test, T maze alternation test, homing test, home retrieval test) but others have assessed other aspects of memory such as memory for objects (object recognition memory test).

**FIGURE 2:**
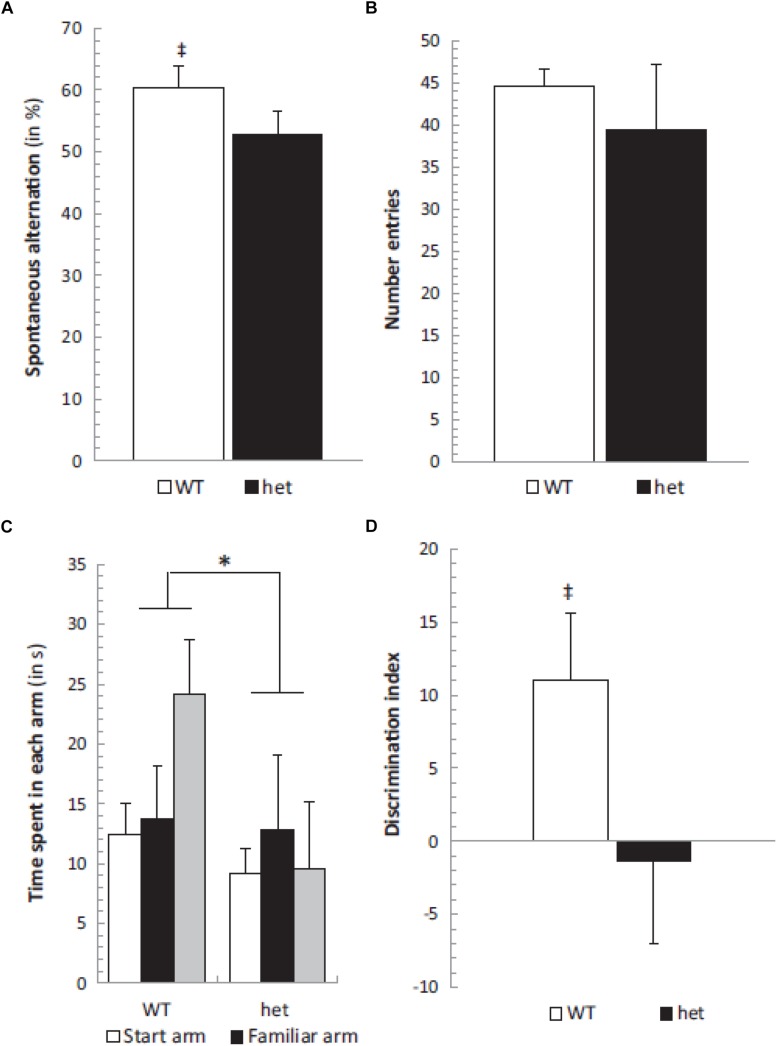
Spontaneous alternation was significantly different from chance level (50%) in the WT group (‡, *p* < 0.05) **(A)** while the number of entries remained similar in both *het*/*het* and WT groups **(B)**. The place recognition test showed a significant group effect between WT and *het*/*het* mice without any specific arm effect or group × arm interaction, but the time spent in the new arm was significantly different in WT and *het*/*het* groups **(C)**. Additionally, the discrimination index was different between groups (^∗^ and ‡, *p* < 0.05), and not different from zero in the *het*/*het* group **(D)**. Reproduced from Machado et al. (2012) with permission.

In order to investigate the effects of selective otolith loss on cognitive and emotional function, Machado et al. (2012) investigated the performance of B6Ei.GL-Nox3het/J mice (“*tilted Het*” mice) in a series of behavioral tests: the Y maze, place recognition, elevated plus maze tests, as well as the rotarod test as a measure of motor control. The mice were 6–8 months of age. In the object recognition test, only the wild-type controls exhibited an exploration time that was significantly greater than chance; however, the discrimination index was not significantly different between the two groups. In the spontaneous alternation test in the Y maze, again, only the wild-type controls but not the *Het* mice showed performance significantly greater than chance (Figure 2). In the place recognition test, wild-type mice spent significantly more time in the new arm compared to *Het* mice (Figure 2). However, the *Het* mice could execute neither the rotarod test nor the elevated plus maze test. The authors noted that the *Het* mice were impaired in spatial memory performance (the spontaneous alternation and place recognition tests) but not non-spatial memory performance (object recognition test).

**FIGURE 3:**
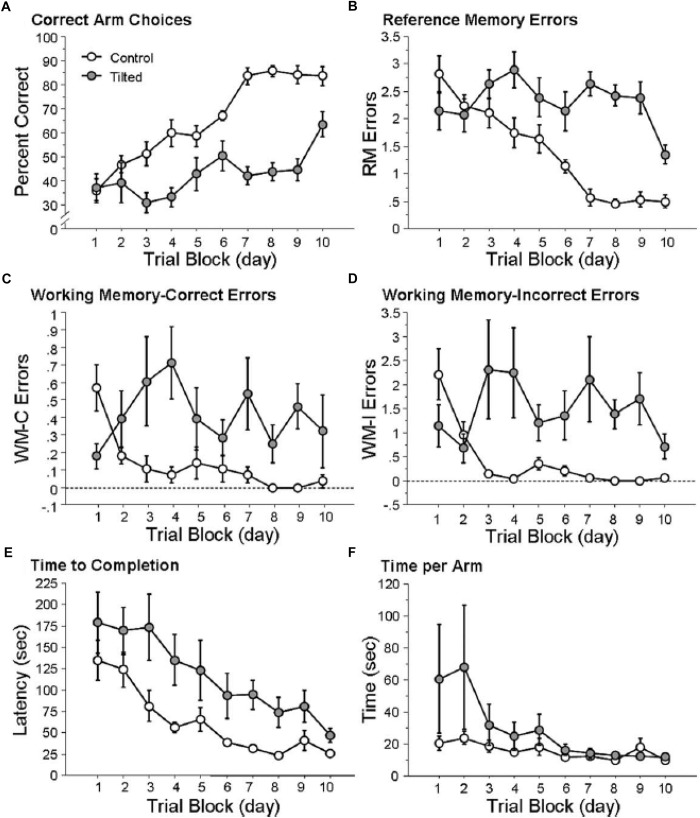
*Tilted* mice were impaired at place learning on the radial arm maze. **(A)** Percentage of correct arm choices increased more rapidly across trial blocks for control mice than for *tilted* mice. **(B–D)** RM, WM-C, and WM-I errors decreased across trial blocks for control mice but not for *tilted* mice. **(E)** Latency to complete the task decreased across trial blocks for both groups. **(F)** Time per arm did not differ between control and *tilted* mice. Mean ± SEM. Reproduced from Yoder and Kirby (2014) with permission.

In order to explore the effects of otolith loss on spatial cognition specifically, Yoder and Kirby (2014) investigated spatial memory in mice lacking otolithic function using the B6.Cg*-Otop1^*tlt/J*^* model, evaluating their performance in both a radial arm maze and a Barnes task in light, compared to heterozygous controls. In this case the status of the mice as –/– or ± was determined using a swim test, since –/– mice are unable to swim at 12 weeks of age. *Tilted* mice were able to learn to navigate to a visible goal in the radial arm maze; however, they exhibited a significantly lower percentage of correct choices and their improvement in performance over trials was significantly worse than the controls (Figure 3). The authors analyzed the specific types of memory errors that were being made. *Tilted* mice made more reference memory (RM) errors (the mouse did not enter an arm that was a correct choice) than controls, and also a slower rate of decrease in these errors over trials (Figure 3). *Tilted* mice also made more working memory (WM) errors than controls (i.e., either they re-entered an arm that was previously baited (WM-C) or an arm that had never been baited (WM-I)). The number of WM-C errors was higher for the *tilted* mice; however, this did not change across trials. For WM-I errors, *tilted* mice exhibited a greater number of them and this was maintained across trial blocks (Figure 3). The *tilted* mice showed a longer latency to complete the radial arm maze task, although the latency decreased across trials and was not significantly different compared to the control group.

In the case of the Barnes maze, the control and *tilted* mice were similar in terms of RM, with both groups employing a serial strategy during the first 2 days of the experiment. By the final day, the control mice were using either a spatial or serial strategy, whereas the *tilted* mice were still using a serial strategy. There were no significant differences in latency, error rates or distance traveled. The fact that the *tilted* mice were impaired in the radial arm maze but not the Barnes maze is very interesting. Impairment in the radial arm maze has been observed previously with complete bilateral vestibular lesions (e.g., Ossenkopp and Hargreaves, 1993; Russell et al., 2003a; Besnard et al., 2012); therefore, these results suggest that similar impairment in this task can be caused by otolith dysfunction alone. Yoder and Kirby (2014) suggested that the impaired performance of the *tilted* mice was a result of them not being able to discriminate between the arms of the radial arm maze, rather than any general memory deficit. The authors point out that rats with complete bilateral vestibular lesions have been reported to be unimpaired in an open field homing task when distal cues were available (Wallace et al., 2002; see also Stackman and Herbert, 2002), indicating that vestibular information is not necessary for the development of spatial memory. They suggest that in tasks such as these as well as the Barnes maze, animals are only required to navigate to a single goal which is defined by its position in relation to landmarks, whereas in the radial arm maze they are required to learn to navigate between multiple goals.

**FIGURE 4:**
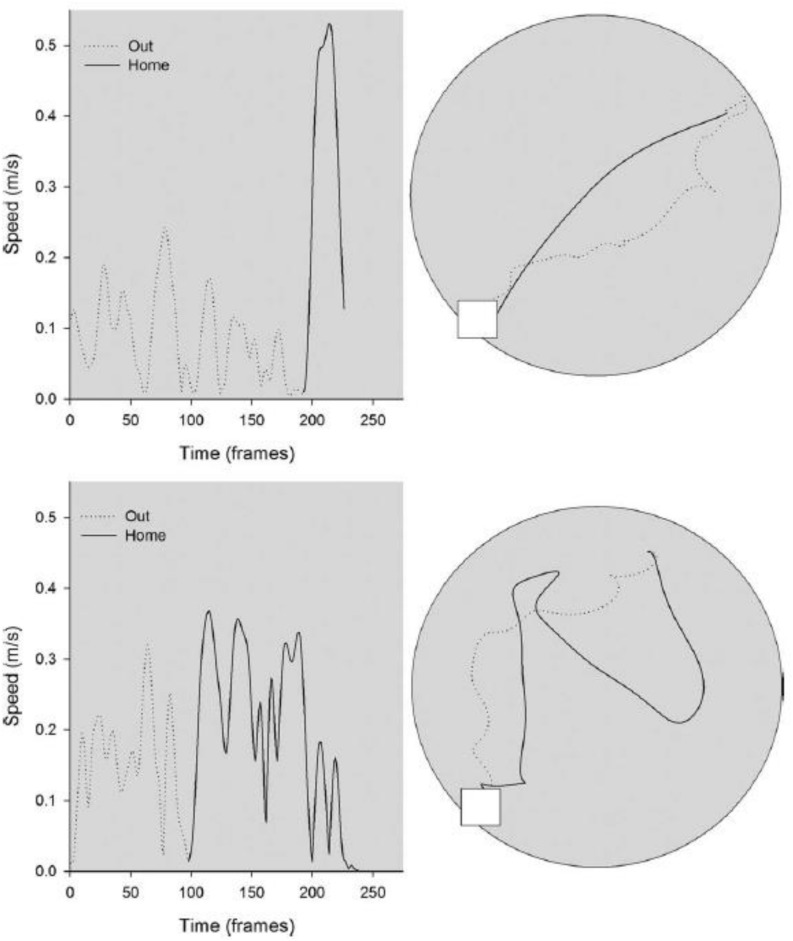
Kinematic **(left)** and topographic **(right)** characteristics are plotted for a representative control **(top)** and *tilted* mouse **(bottom)** on the homing task in darkness. The outward path (gray dashed line) is circuitous for both mice; the homeward segment (black line) is relatively direct for the control mouse, but is more circuitous for the *tilted* mouse. Reproduced from Yoder et al. (2015) with permission.

In a further study of the effects of otolith loss on spatial cognitive function, Yoder et al. (2015) studied the homing ability in the B6.Cg-*Otop1*^*tlt/J*^ model, in both darkness and light, compared to heterozygous controls. The mice were classified as –/– or ± based on the same swim test, used by Yoder and Kirby (2014) and were 3–8 months of age at the beginning of testing. They found that in darkness, in the absence of visual information, the *tilted mice* made more short duration stops while they were on the outward journey to find food, and, once they had found it, their route home was more circuitous than for heterozygous control mice (see Figure 4). When this was quantified, the *tilted mice* exhibited greater heading error and journey duration than the controls. When they were tested in light, while the *tilted* mice still exhibited a more circuitous journey than the controls, their heading error and journey duration were similar. Blankenship et al. (2017) have also reported that B6.Cg-*Otop1*^*tlt/J*^ mice, at 12 weeks of age, exhibit significantly greater circuitous exploratory movements in darkness, with greater changes in heading direction, compared to controls. The *tilted* mice also exhibited greater heading changes in light, and the authors concluded that they were using visual cues to compensate for the lack of otolithic information about self-motion.

**FIGURE 5:**
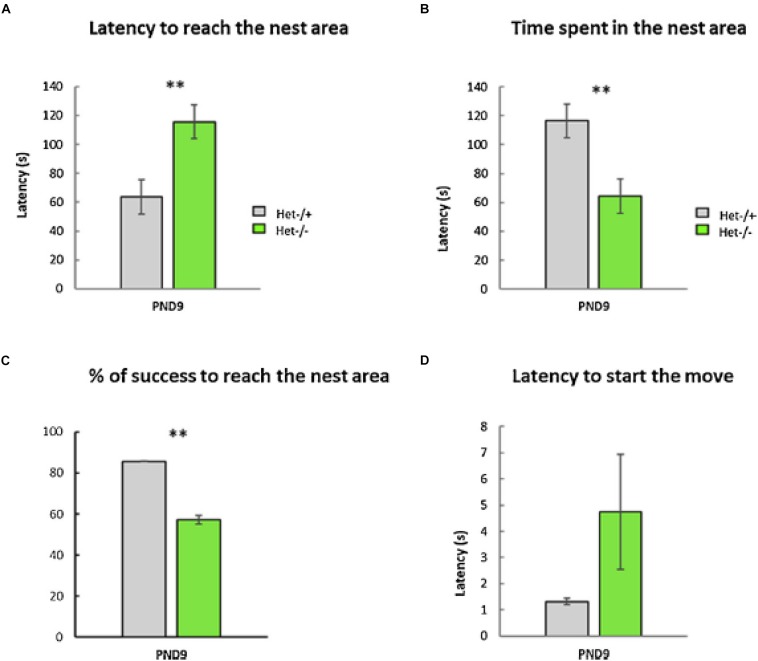
The Home Retrieval test performed at PND9. No differences were detected between males and females; therefore, data were collapsed across sex (*p*-value > 0.05, *n* = 25 *Het*–/– versus 36 *Het–/*+). Two aberrant values were excluded in this test. **(A)** The *Het*–/– group needed significantly more time to reach the nest area compared to the *Het–/*+ control group (“Latency to reach the nest,” Mann–Whitney test, *p* ≤ 0.01, ^∗∗^, data ± sem). **(B)** The time spent in the nest area was significantly lower in the *Het*–/– group compared to the *Het–/*+ control group (Mann–Whitney test, *p* ≤ 0.01, ^∗∗^, data ± sem). **(C)** The otolith-deficient *Het*–/– pups showed a lower percentage of success reaching the nest area compared to *Het–/*+ mice (Chi^2^-tests for equality of proportions, *p* ≤ 0.01, ^∗∗^, data ± sem). **(D)** Conversely, the latency before the first movement reaction of pups (Mann–Whitney test, *p* > 0.05, ns, data ± sem) was similar in both groups (*Het*–/–, *Het–/*+). Reproduced from Le Gall et al. (2019) with permission.

As part of a larger study of the effects of otolith loss on sensorimotor development, Le Gall et al. (2019) evaluated *tilted Het* mice in a home retrieval test on post-natal day 9, in order to test their spatial memory. Their phenotype was confirmed by PCR and they were compared to heterozygous controls. The home retrieval test is based on the natural tendency of blind pups to find their way back to the safety of their nest, using olfactory information. Le Gall et al. (2019) found that the *Het* mice exhibited a significantly longer latency to reach the nest area, they spent significantly less time in that area and were significantly less successful in reaching it, compared to the controls (Figure 5). Although this task involved an element of spatial memory in the form of spatial olfactory guidance, the *Het* mice were found to exhibit significant delays in sensorimotor reflex development and ultrasonic communication; maternal care was, however, normal.

In the most recent study of the effects of otolith loss on cognitive and emotional function, Manes et al. (2019) used the *mergulhador (mlh)* otoconia-deficient mouse model, to investigate the effects of otolith deficiency on open field activity, performance in the novel object recognition test, the T maze alternation test, the elevated plus maze test as well as neurochemical activity in various brain regions. The *mlh* mutation was confirmed by genetic analysis and the mice were 8 weeks of age at the beginning of testing. Compared to *BALB/c* mice, the mutant mice exhibited reduced locomotor behavior and rearing, with increased grooming in the open field maze. Motor coordination on the wooden beam test was reduced, and immobility time increased in the tail suspension test. In addition, the mutant mice displayed reduced auricular reflexes and responsiveness to touch. Although the T maze performance was significantly reduced for the mutant mice, there were no significant differences for the novel object recognition test or the elevated plus maze test.

In order to explore the neurochemical basis of the effects of otolith loss, the authors conducted a neurochemical investigation of the cerebellum, anterior part of the cortex, striatum and hippocampus, following decapitation, and analyzed the levels of dopamine (DA), noradrenaline (NA) and their metabolites [4,4-dihydrophenylacetic acid (DOPAC) and homovanillic acid (HVA)], in addition to serotonin and its metabolite, 5-hydroindole, 3-acetic acid (5HIAA). The results were complex (see Table 2 in Manes et al., 2019), but suggested an increase in DA turnover in the hippocampus with a concomitant decreased turnover of DA and reduced NA activity in the frontal cortex. The authors suggested that these neurochemical alterations could be responsible for the impaired response of the *mlh* mice in the T maze task.

Taken together, although there is still a small number of studies, most of this evidence suggests that the loss of otolith function results in an impairment of spatial memory.

## Evidence for Otolithic Involvement in Spatial Memory in Humans

The studies described in the previous section provide evidence that the absence of otolith function from birth in mice, appears to cause spatial memory deficits. Is there also a relationship between otolithic dysfunction and spatial memory in humans and can this develop later in life? Over the last several years, a number of epidemiological studies have been published on the relationship between vestibular function, aging and spatial memory (see Agrawal et al., 2019 for a review). Many of these studies have related spatial memory specifically to cervical vestibular-evoked myogenic potentials (cVEMPs), a vestibulo-spinal reflex that originates in the saccular part of the otoliths (see Curthoys et al., 2017 for a review; see Figure 1). Some studies have also assessed ocular VEMPs (oVEMPs), which are generated by the utricle (see Figure 1).

Harun et al. (2016) studied older subjects with mild cognitive impairment (MCI, *n* = 15) or Alzheimer’s Disease (AD, *n* = 32) and measured their cVEMPs and oVEMPs, comparing them with age-, sex-, and education-matched controls (*n* = 94) from the Baltimore Longitudinal Study of Aging (BLSA). Surprisingly, the authors found that bilaterally absent cVEMPs were associated with a more than 3-fold increased odds of AD (OR 3.42, 95% CI 1.33–8.91). However, there were no significant differences in vestibulo-ocular reflex (VOR) gain between the groups. The same group have reported that patients with MCI (*n* = 22) or AD (*n* = 28) and saccular dysfunction, measured using cVEMPs, made significantly more errors on the Money Road Map Test (MRMT) of spatial cognition compared to patients without vestibular impairment. AD patients who were spatially impaired exhibited a significantly greater prevalence of vestibular loss (Wei et al., 2018). Wei et al. (2017) examined driving ability in 21 patients with MCI and 39 with AD and related these data to saccular function. Using multiple regression, they found that patients with MCI or AD, who also had bilateral saccular impairment, had a significant increase in the odds of experiencing driving difficulty (OR 12.1; 95% CI 1.2, 117.7), compared to MCI or AD patients with normal saccular function. The patients’ performance on the MRMT suggested that the driving difficulty might be attributable to spatial memory deficits [see also Anson et al. (2019) and Pineault et al. (2019) for the most recent evidence].

Xie et al. (2017) studied the performance of young (<55 years) and older (≥55 years) subjects in the Triangle Completion Task (TCT), which tests spatial navigation ability, and related it to vestibular function. They found that loss of otolith function, as measured using the cVEMP, as well as loss of function of the semi-circular canals, was related to poor performance in the TCT in older subjects. Kamil et al. (2018) studied 103 subjects from the BLSA, measuring cVEMP function and relating it to MRI analysis of the hippocampus. They found that decreased saccular function, defined as a lower cVEMP amplitude, was significantly related to a lower average hippocampal volume. This result suggests the possibility that age-related impairment of spatial memory, as well as impairment of saccular function, may be related to a decrease in hippocampal volume. In their most recent study, Wei et al. (2019), studied 26 patients with MCI, 51 with AD and 295 matched control subjects and evaluated their cVEMPs, oVEMPs and VOR gain. They found that with increased cognitive impairment, there was a significant decrease in cVEMP and oVEMP responses. The VOR gain was lowest in MCI patients, followed by AD patients and then control subjects. Using logistic regression, they found that abnormal cVEMPs were associated with a 3-fold increased odds of MCI and a 5-fold increased odds of AD, and that abnormal oVEMPs were associated with an almost 4-fold increased odds of MCI and an over 4-fold increased odds of AD. However, VOR gain was not significantly related to the probability of having AD. See Addendum for further references published while this paper was in press.

Vestibular loss has often been associated with depersonalization/derealization (DD) symptoms in humans (see Besnard et al., 2016 for a review). These symptoms include feelings of déjà vu, of time passing slowly, being “spaced out,” difficulty concentrating, thoughts seeming blurred, one’s body feeling strange and not feeling in control of one’s self. In an experimental study, Aranda-Moreno and Jáuregui-Renaud (2016) used unilateral centrifugation in order to stimulate the utricle of 100 healthy subjects, who then completed a depersonalization/derealization (DD) questionnaire. The subjects reported symptoms such as “Body feels strange or different in some way” (56%) and “Time seems to pass very slowly” (55%). The authors concluded that utricular stimulation induces DD symptoms in healthy subjects.

## Effects of Otolith Loss on Neural Activity

Given the evidence that loss of the otoliths in mice or otolith dysfunction in humans is associated with spatial memory deficits, it was logical to consider what effect the loss of otolithic information might have on the neural pathways subserving spatial memory. An obvious place to look was head direction cells in the thalamus and elsewhere, and place cells in the hippocampus, since they are known to be critical for spatial memory. Although it is clear that vestibular information is transmitted to the thalamus and hippocampus via polysynaptic pathways (see Hitier et al., 2014 for a review), the pathways through which specifically otolithic information might be communicated, are unclear.

Few studies have electrically stimulated the otoliths selectively in rodents, in order to determine where otolithic information might be represented in structures such as the hippocampus. Cuthbert et al. (2000) reported inducing field potentials in CA1 by selective electrical stimulation of the utricle in guinea pig, and furthermore they found that they could still induce field potentials when the stimulus amplitude was too low to evoke the VOR. Hitier et al. (2019) have also reported that they could induce local field potentials in many areas of the hippocampus by selective electrical stimulation of the utricle and saccule in anesthetized rats.

### Thalamic Studies

**FIGURE 6:**
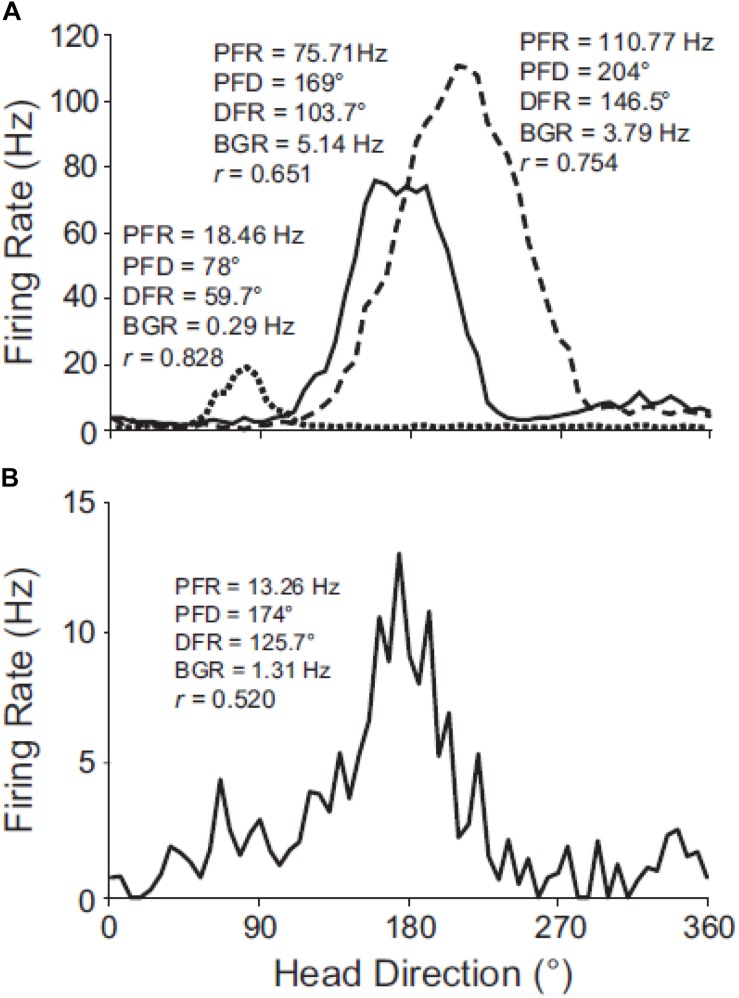
Directional tuning curves of HD cells in C57BL/6J and *tilted* mice. **(A)** Robust directional tuning of three classic HD cells recorded simultaneously on different electrodes from the same C57BL/6J mouse. Peak firing rate (PFR), preferred direction (PFD), directional firing range (DFR), and background firing rate (BFR) are unique to each HD cell. **(B)** Tuning curve of representative HD cell recorded from a *tilted* mouse. Note the variable background firing rate (outside of the directional firing range) for the *tilted* HD cell, which contrasts with the relatively uniform background firing rates of C57BL/6J HD cells. Rayleigh’s *r* is noted for each cell. Reproduced from Yoder and Taube (2009) with permission.

Apart from selective otolithic stimulation, another way of studying the effect of the otoliths on brain regions which are spatially responsive, is to employ the same otolith-deficient mouse models that were described in the behavioral studies. The effects of otolith deficiency on the neural substrates of spatial navigation and memory were first studied by Taube (2011) and Yoder and Taube (2014) (for reviews). Stackman and Taube (1997) had demonstrated that the normal firing properties of head direction (HD) cells in the anterior thalamus of the rat, required vestibular input in general. Yoder and Taube (2009) used *Otop1*^*tlt*^ mice to investigate the effects of otolith deficiency on HD neurons in the anterodorsal thalamus. The firing properties of cells in the *tilted* mice were not as robust as those in wild-type *C57BL/6J* mice (see Figure 6) and exhibited significant degradation across trials. In similar experiments, Calton and Taube (2005) demonstrated that when rats performed inverted locomotion, 47% of HD cells in the anterodorsal thalamus showed no directional firing selectivity, even though they had demonstrated this on the walls before they were inverted. During the process of inverted locomotion, the action of gravity on the saccule should have been the opposite of what it is normally.

Some studies of HD cells have been conducted in microgravity, where the normal stimulation of the otoliths by gravitational acceleration is absent. Taube et al. (2004) studied anterodorsal thalamic HD cells during the zero gravity phase of parabolic flight. They found that HD cells maintained their directional tuning during both the zero gravity and hypergravity phases of the flight when the rat was positioned on the floor, but that when the rat climbed the wall or moved upside down on the ceiling in zero gravity, they lost their directional firing and exhibited an increase in spontaneous firing rate. There was no significant difference for the peak firing rate and baseline firing rate for 1-G and 0-G on the floor, but significant decreases for the signal-to-noise ratio and information content of the HD firing. The cells exhibited a significantly reduced directionality score in 0-G, on average, by 25.8%, although they still exhibited directional tuning. While on the wall in 0-G, the HD cells that were recorded exhibited little directional preference. Compared to 0-G on the floor, all 7 HD cells that were recorded were found to lose their directional tuning when on the ceiling in 0-G: there were significant decreases in the signal-to-noise ratio, information content and the directionality score, with a significant increase in baseline firing rate. The authors speculated that during 0-G, the rats may have been experiencing visual reorientation illusions (VRIs).

The fact that both HD cell function and spatial memory are degraded by the loss of otolithic information, does not prove that there is a causal connection between the two. The data presented by Taube et al. (2004) would suggest that rats might have difficulty learning and remembering a spatial memory task while upside down. Valerio et al. (2010) investigated whether rats that were inverted on a circular platform suspended from the ceiling, could learn to navigate to an escape hole from a start point. They found that none of the animals trained from 4 start points were able to reach the criterion, even following 29 training sessions; by contrast, all of the rats trained from one start point reached the criterion after a mean of 5.9 training sessions. Further experiments suggested that rats used distal visual landmarks when navigating from 1 or 2 start points, but that their performance was degraded when they needed to navigate to the escape hole from a novel start point. The authors suggested that in the upside down situation, the rats do not learn to reach a certain place, but rather learn a series of separate trajectories to reach the target.

One of the optimal ways of studying the effects of the loss of the otolithic response to gravitational acceleration, without using lesions or a mutant mouse model, is to investigate the behavior of mice in space. Ronca et al. (2019) have recently reported a study of mice aboard the International Space Station (ISS). Following a 4 day transit from Earth to the ISS, they analyzed the behavior of 16- and 32-week old female mice. They found that the young flight mice engaged in more physical activity than controls on Earth, but that 7–10 days following the launch, the younger, but not older mice, exhibited a form of “race-tracking behavior” which was expressed as circling. The significance of this result is unclear. Interestingly, however, circling and hyperactivity are common symptoms of bilateral vestibular loss in rats and mice (see Stiles and Smith, 2015, for a review).

### Hippocampal Studies

**FIGURE 7:**
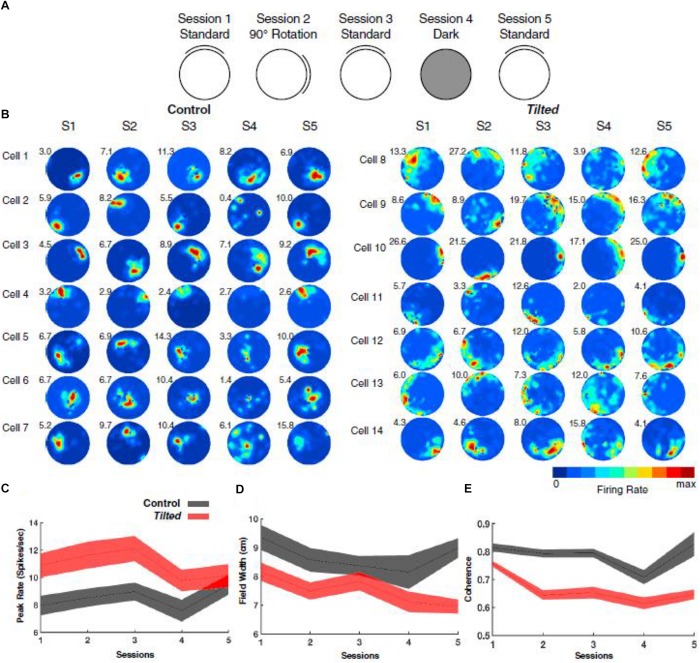
Overview of experimental design, place-cell examples, and basic firing characteristics. **(A)** Depiction of recording procedure across five recording sessions: Session 1, cue card was positioned in the standard north position; Session 2, cue card was rotated 90° clockwise or counterclockwise from the standard location; Session 3, cue card was returned to the standard location; Session 4, cue card was removed, and overhead lights were extinguished; and Session 5, white cue card was replaced at the standard location, and lights were turned on. **(B)** Representative place cells from control (cells 1–7) and *tilted* (cells 8–14) mice over five sessions. Numbers at the top left of each rate map represent peak firing rate (Hz). **(C)** Plot showing the peak firing rate (spikes/second) for each place cell recorded in *tilted* and control mice with values from all sessions included. **(D)** Plot showing the field width (cm) for each place cell recorded in *tilted* and control mice with values from all sessions included. **(E)** Plot showing coherence measures for each place cell recorded in *tilted* and control mice with values from all sessions included. **(C–E)** Shaded error bars represent SEM. Reproduced from Harvey et al. (2018) with permission.

**FIGURE 8:**
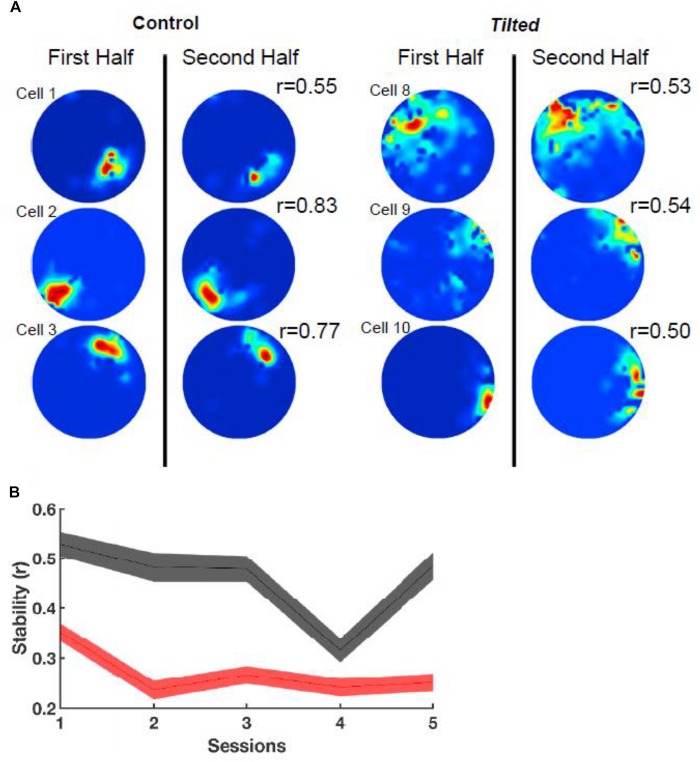
Intra-session stability. **(A)** Rate maps depict the stability (r) between the first and second half of the first session using six example cells from Figure 1. Note that *tilted* cells appear to be less stable than control cells. **(B)** Stability between groups over all sessions. Note that *tilted* mice have much lower stability compared to control mice for all sessions. Red, *tilted*; black: control; shaded lines represent SEM. Reproduced from Harvey et al. (2018) with permission.

In addition to thalamic HD cells, it is also of interest to determine the effects of the lack of graviception by the otoliths, on place cells in the hippocampus. Place cells in the hippocampus have been demonstrated to rely on input from the vestibular system (e.g., Stackman et al., 2002; Russell et al., 2003b); however, once again, the extent to which the otoliths are specifically implicated has been unclear. Knierim et al. (2000) had recorded hippocampal place cells on the Neurolab Spacelab Mission and reported, in a brief letter, that normal place fields could be obtained by day 9 of the space flight. However, the more comprehensive NASA report did indicate that abnormal place fields were observed earlier in the flight, for example, day 4 (Knierim et al., 2003). The only study to address this question using otolith-deficient mice is by Harvey et al. (2018), who recorded hippocampal place cells in B6.Cg-*Otop1tlt/j tilted* mice (Jackson Laboratories, Bar Harbor, ME). They investigated place cells across five sessions (standard, cue rotation, standard, dark, standard) and found that those in *tilted* mice exhibited higher peak firing rates, with lower spatial coherence and smaller fields, compared to controls (see Figure 7); on the other hand, the average firing rates and spatial information content, were similar between the groups. The place fields from *tilted* mice were also less smooth than for controls and were concentrated near boundaries. Although hippocampal place cells are modulated by theta rhythm and theta has been reported to be degraded following complete bilateral vestibular loss (e.g., Russell et al., 2006), place cell activity was still modulated by theta in *tilted* mice. The place fields from *tilted* mice exhibited lower intra-trial stability than controls (Figure 8). It had been suggested in previous studies that otolithic information might be especially important for accurate spatial navigation performance in darkness in homing tasks and radial arm mazes (Yoder and Kirby, 2014; Yoder et al., 2015).

One of the most recent studies linking hippocampal and otolithic function in mice is by Kompotis et al. (2019). They observed that rocking mice at 1 Hz increased the amount of time spent in non-rapid eye movement sleep by accelerating the onset of sleep and shortening waking episodes, and that during active wakefulness, the theta EEG (6–10 Hz) moved toward slower frequencies. They used *Otop1^*tlt/tlt*^ tilted* mice to test whether the otoliths were involved in this phenomenon and found that they were insensitive to rocking at 1 Hz and did not exhibit the same changes in theta EEG.

Although some would consider that galvanic vestibular stimulation (GVS) might be useful to probe the effects of otolith stimulation on the brain (e.g., Cohen et al., 2012), the evidence that it affects “mainly” the otoliths, is divided, and there is evidence that it also affects the semi-circular canals (see Curthoys and MacDougall, 2012, for a review). Therefore, GVS studies will not be discussed here.

## Conclusion

These studies suggest that the otolithic part of the peripheral vestibular system, the utricle and saccule, make some contribution to spatial learning and memory in tests such as the radial arm maze, Y maze and homing tasks, but not the Barnes task or non-spatial memory tests such as the object recognition test or elevated plus maze task. The link between otolithic function and spatial memory is reinforced by human epidemiological studies in which it has been shown that saccular function, as measured using cVEMPs, is a predictor of the odds of having poor spatial memory, AD, and a smaller hippocampal volume. A link between hippocampal volume and vestibular function was first reported by Brandt et al. (2005), in a study in which patients with bilateral vestibular loss were found to have a bilateral atrophy of the hippocampus of approximately 17%. However, it was not clear from this study whether the otoliths were important in this relationship. The neurophysiological studies in otolith-deficient mice indicate that otolithic function is important for the normal function of both thalamic HD cells and hippocampal place cells, although HD cells can maintain directional tuning as long as they are on the floor in microgravity (Taube et al., 2004) and place cells can still develop place fields in microgravity (Knierim et al., 2000, 2003). Taube et al. (2004) suggested that the loss of directional firing of HD cells when they are on the walls or ceiling in 0-G may be a correlate of Type II/III spatial disorientation in humans, in which the subject is consciously aware of their disorientation and tries to correct it (Type II) or is so disoriented that they are incapacitated (Type III), respectively. Clearly, the loss of HD cell tuning is due to more than the loss of a gravitational signal (Taube et al., 2004).

**FIGURE 9:**
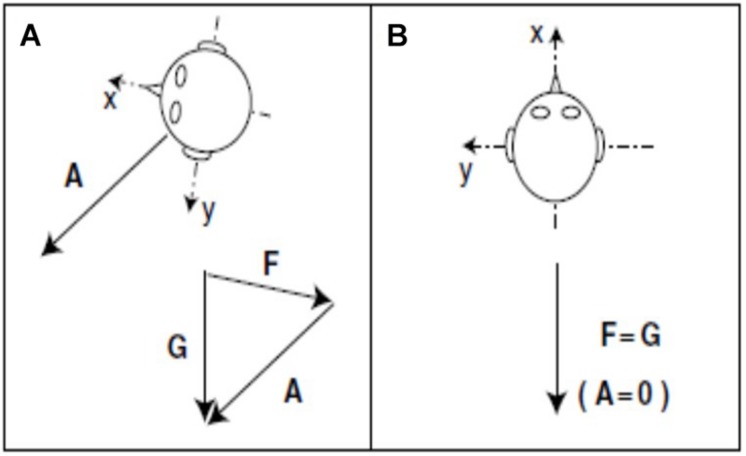
During acceleration on Earth, gravity (G) minus linear acceleration (A) yields specific gravito-inertial force (GIF, F). Different combinations of gravity and linear acceleration can yield the same measured GIF. **(A)** GIF equals gravity minus linear acceleration (F = G – A or F + A = G). **(B)** GIF equals gravity alone. Since the measurement of GIF relative to the head is identical for these two situations, additional information is required for the nervous system to determine how much of the measured GIF is due to gravity and how much is due to linear acceleration.

In the case of the otolith-deficient mice, which is the easiest form of otolith manipulation to interpret, an interesting question is whether the sensation of linear acceleration by gravity is more important for spatial memory than the sensation of other kinds of linear acceleration, such as translation in the anterio-caudal or medio-lateral planes? In fact, the “tilt-translation illusion” demonstrates that the otoliths cannot distinguish between tilting of the head with respect to gravitational vertical and any other kind of linear acceleration that would induce the same stimulation of the otoliths (“Einstein’s Equivalence Principle,” Merfeld et al., 1999; Figure 9). Therefore, the brain must use information from the semi-circular canals and other sensory information to disambiguate tilt from translation (Merfeld et al., 1999; Straka et al., 2016). Since every form of sensory stimulation on Earth must be interpreted in terms of the framework of gravitational acceleration, it may be that otolithic stimulation is essential for normal spatial learning and memory. This is consistent with the evolutionary age of the otoliths. Although a number of studies have attempted to neutralize the influence of the vestibular system by fixing the heads of rats or mice and recording from HD, place or grid cells (e.g., Harvey et al., 2009; Ravassard et al., 2013; Aghajan et al., 2015; Acharya et al., 2016), it is debatable whether these studies have done any more than “minimize” vestibular stimulation, because the head will still be subject to linear acceleration by gravity.

Yoder and Taube (2009) speculated that part of the explanation for the broader tuning curves of HD cells in *tilted* mice in light, might be degraded visual perception of visual cues due to an impaired VOR (“oscillopsia”). This certainly seems possible in light, however, the greater reductions in HD cell directional preference in darkness indicates that oscillopsia is not necessary for their impaired function. The authors suggested that loss of otolithic function may impair velocity storage, which provides a prolonged representation of the angular velocity signal in the brain and may be necessary for accurate spatial navigation. Although the otoliths do not encode angular acceleration themselves, they are known to be important in velocity storage (e.g., Angelaki et al., 1992; Koizuka et al., 1996; Mittelstaedt and Mittelstaedt, 1996; Wearne et al., 1999). Recent studies using *Otop1* mice show that although the otoliths do not appear to contribute to the baseline angular velocity of the VOR in mice, they are necessary for its adaptation to changes in gravitational stimulation (Khan et al., 2019a, b). As Shinder and Taube (2014) noted, the fact that the otoliths are known to be important for HD cell firing, suggests that linear acceleration information from the otoliths and rotational acceleration information from the semi-circular canals, must converge by the time they reach the thalamus. Indeed, Curthoys and Markham (1971) demonstrated the convergence of otolith and semi-canal signals at the level of the medial vestibular nucleus.

Taken together, the evidence to date suggests that the otolithic organs of the vestibular system are important for normal spatial learning and memory. Although the mechanisms by which they contribute to these processes are yet to be fully elucidated, the saccule and utricle appear to contribute to the development and maintenance of normal HD and place cell signals. This may be due to the evolutionary age of this part of the vestibular system and the fact that velocity storage has become an important part of the way that the brain encodes its place in space (Yoder and Taube, 2009).

## Author Contributions

PS conceived the study and wrote the manuscript.

## Conflict of Interest

The author declares that the research was conducted in the absence of any commercial or financial relationships that could be construed as a potential conflict of interest.
